# Genome-wide analysis of primary peripheral blood mononuclear cells from HIV + patients-pre-and post- HAART show immune activation and inflammation the main drivers of host gene expression

**DOI:** 10.1186/2052-8426-2-11

**Published:** 2014-04-03

**Authors:** Viviane N da Conceicao, Wayne B Dyer, Kaushal Gandhi, Priyanka Gupta, Nitin K Saksena

**Affiliations:** Centre for Virus Research, Westmead Millennium Institute, Westmead Hospital, University of Sydney, Darcy Road, Sydney, Westmead, NSW 2145 Australia; Retroviral Genetics Division, Centre for Virus Research, Westmead Millennium Institute, Sydney, Westmead, NSW 2145 Australia

## Abstract

**Background:**

Although the host gene expression in the context of HIV has been explored by several studies, it remains unclear how HIV is able to manipulate and subvert host gene machinery before and after highly active antiretroviral therapy (HAART) in the same individual. In order to define the underlying pharmaco-genomic basis of HIV control during HAART and genomic basis of immune deterioration prior to HAART initiation, we performed a genome-wide expression analysis using primary peripheral blood mononuclear cells (PBMC) derived from 14 HIV + subjects pre-highly active antiretroviral therapy (HAART) (time point-1 or TP1) with detectable plasma viremia and post-HAART (time point-2 or TP2) with effective control of plasma viremia (<40 HIV RNA copies/mL of plasma).

**Methods:**

Genomic RNA extracted from the PBMCs was used in microarray analysis using HT-12V3 Illumina chips. Illumina®BeadStudio Software was used to obtain differentially expressed (DE) genes. Only the genes with *p value* <0.01 and FDR of <5% were considered for analysis. Pathway analysis was performed in MetaCore™ to derive functional annotations. Functionally significant genes were validated by qRT-PCR.

**Results:**

Between TP1 and TP2, 234 genes were differentially expressed (DE). During viremic phase (TP1), there was an orchestrated and coordinated up-regulation of immune, inflammation and antiviral genes, consistent with HIV infection and immune activation, which comprised of genes mainly involved in antiviral action of interferons and their signalling. In contrast, the therapy-mediated control phase (TP2) showed systematic down-regulation of these pathways, suggesting that the reduction in plasma viremia with HAART has a considerable influence on reducing the immune activation, thereby implying a definitive role of HIV in subverting the human gene machinery.

**Conclusions:**

This is the first study to show the evidence for the differential regulation of gene expression between the untreated and treated time points, suggesting that gene expression is a consequence of cellular activation during plasma viremia. Affirmation to these observations comes from down-modulation of genes involved in cellular activation and inflammation upon initiation of HAART coinciding with below detectable levels of plasma viremia.

**Electronic supplementary material:**

The online version of this article (doi:10.1186/2052-8426-2-11) contains supplementary material, which is available to authorized users.

## Background

The natural history of HIV is tightly governed by the plasma viral load and T cell modulation in the absence of HAART, which results in massive destruction of CD4+ T cells by multiple mechanisms, leading to T cell exhaustion. HAART leads to dramatic decrease in HIV RNA levels and aids in reducing incidence of opportunistic infections and co-morbidities, mortality rates and stopping secondary transmission [1, 2].

It is apparent that HIV not only targets CD4+ T cells, but also has the ability to infect a variety of blood leukocytes [1, 3–5]. This shows that HIV has the inherent ability to subvert and manipulate the host gene machinery at the transcriptomic level [6–8], thereby having considerable influence on the cell morphology, gene expression, and metabolism. Increased gene expression changes have also been associated with increased viral loads in viremic patients. Nonetheless the global effects of viral infection on host cell gene expression patterns pre- and post- antiretroviral therapy in HIV + patients, still remain poorly understood [9]. Recently, the high-density genome-wide microarrays have greatly facilitated the understanding of genomic basis of host-pathogen and pharmaco-genomic interactions [9, 10].

Following the introduction of HAART, most HIV + patients who adhere to treatment show a good response, defined by a decrease of plasma viral load (pVL) to undetectable levels and an immunological reconstitution with a significant increase of CD4+ T cell levels from baseline values leading to prolonged survival [11]. The immune reconstitution fails under the value of 200 CD4+ T cells/ml, which is considered as a critical threshold, and this occurs in 5–27% of patients receiving HAART [12, 13]. In fact, CD4 + T-cell counts persistently <250 cells/ml, or a percentage of CD4+ T cells <17%, has been considered a sign of poor immune reconstitution [14]. In our study, all our patients before the start of therapy showed 300 CD4+ T cells/mL of blood. Therefore, we hypothesize that successful HAART treatment in responders can guide us to find pharmaco-genomic basis of immunological reconstitution and identify key immune pathways that cooperate during immune reconstitution upon effective therapy. To better characterize genomic events occurring *in vivo* before and after therapy in HIV + patients, we have used genome-wide analysis using cDNA microarrays on frozen primary *ex-vivo*-derived (uncultured) PBMCs from HIV + patients at two times points- before (being referred to as TP1 - with detectable plasma viremia) and after the initiation of HAART (being referred to as TP2 with below-detectable <20 copies HIV RNA/mL plasma). The TP2 samples were collected after 2-3 years apart from the initiation of HAART. All patients showed rise in CD4+ T cell counts upon suppressive HAART and reduction in plasma viremia to below detectable levels of HIV RNA and were therefore, termed responders.

In the past, studies using paired design on HIV infected patients (before and after HAART) have shown that several genes are differently expressed between the patients. They also show that the treatment has a limited effect on gene expression *in vivo*[15, 16]. In this study, we show for the first time a genome-wide snapshot of transcriptomic expression demonstrating that the up-regulation of genes involved in innate and adaptive immunity, inflammation, apoptosis and antiviral functions were unique to pre-therapy with detectable plasma viremia, while their down-regulation coincided with complete suppression of plasma virus to below detectable levels (<40 copies/ml plasma) post-HAART. This distinct regulation of host genes pre-and post-HAART underpins the significant role of genes involved in immune activation and inflammation in driving host gene expression during active HIV infection.

## Methods

### RNA extraction

For the total RNA extraction, from the frozen PBMCs the cells were first lysed RNA was extracted as per manufacturer’s instructions (Qiagen RNeasy purification kit, Germany). An on-column digestion of DNA during RNA purification was performed to purify the samples. The DNase was efficiently removed in subsequent wash steps. Total RNA quality was assessed and its concentration was measured using the Agilent RNA 6000 series II Nano kit (Agilent Technologies, CA, USA) through Agilent 2100 Bioanalzer as per manufacturer’s protocol. All RNA integrity numbers considered suitable for microarray were 7 or higher for all samples analysed.

### Genome-wide microarray

Total RNA was reverse transcribed to synthesize the first strand of cDNA, followed by a second-strand synthesis. Double-stranded cDNA was then transcribed and amplified *in vitro* to synthesize biotin labelled complementary mRNA (cRNA). cRNA amplification and labeling with biotin were performed using Illumina TotalPrep RNA amplification kit (Ambion, Inc., Austin, USA) with 250 ng total RNA as input material. cRNA yields were quantified with Agilent Bioanalyzer. Seven hundred and fifty nanogram of cRNA sample was hybridized on a HumanHT12 V3 Expression Bead Chip (Illumina, Inc., CA, USA). The chips were stained with streptavidin-Cye3 conjugate and scanned using an Illumina BeadArray Reader (Illumina, Inc).

### Differential gene expression analysis

Preliminary gene expression analysis was first performed using Bead Studio software version 3, followed by a detailed analysis in BRB Array Tools. Data was normalized with cubic spline function in order to minimize variation due to non-biological factors. The average signal intensity for each gene was measured using Beadstudio v3. Around 24, 000 genes were selected for differential expression analysis with detection P-value less than 0.01 [17].

**Figure 1:**
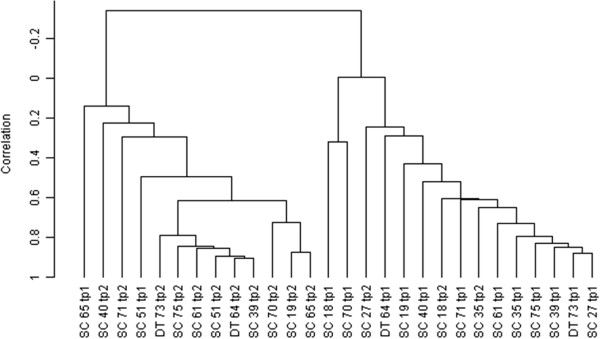
**Clustering analysis of global gene expression profiles in PBMC at time points TP1 and TP2 of the patients enrolled in the study.** Similarities in the gene expression patterns among individuals were evaluated and visualized with BRB ArrayTools. The Y-axis shows the correlation and the X-axis shows the time points of each patient. The algorithm used is named Correlation, which computes the Pearson correlation using a 1-r distance measure. The distance on the X axis represents the similarity relationships among samples.

Following normalization of the entire chip data, which covered more than 25,000 annotated human genes and more than 48,000 probes covering RefSeq and UniGene annotated genes, we performed clustering analysis in BRB-Array Tools software. The statistical significance test used by the BRB-Array software on the 234 DE genes was Pearson’s centred correlation and the average linkage method was implemented to obtain the Figure 1.

The data acquired from the Illumina Microarray BeadChip, was further used for obtaining the DE list in Illumina®BeadStudio Data Analysis Software. Further, this DE gene list was analysed using the BRB-ArrayTools software installed as an Excel package in order to perform a variety of pre-processing steps including computing probe-set expression summaries, normalization, filtering and calculating quality control indices [18–20]. Quantile normalization was performed to diminish the biological errors due to false positives, to see more clearly the systematic biological differences between the samples and to compensate for systematic technical differences between chips. On the BRB-Array Tools software, the Significance Analysis of Microarrays (SAM) was also performed to identify the genes with statistically significant changes in expression by assimilating a set of gene-specific t-tests. During this process, each gene is assigned a score on the basis of its change in gene expression relative to the standard deviation of repeated measurements for that gene. Genes with scores greater than a threshold are deemed potentially significant. The percentage of such genes identified by chance is the false discovery rate (FDR). To estimate the FDR, nonsense genes were identified by analysing permutations of the measurements. The thresholds were adjusted to identify smaller or larger sets of genes, and FDRs were calculated for each set [21]. In this part of the study we used a *FDR* of <0.05 with *p-value* of <0.01 and -1.10 ≤ fold change ≥ 2.85, so the genes that received a fold-change value higher or equal to -1.10 were appointed down-regulated and the genes that received a fold-change minor or equal than 2.85 were up-regulated. The cut off for the fold change was just a consequence of the selection of FDR and p-value.

Further, the differentially expressed genes between TP1 and TP2 were identified using MeV (MultiExperiment Viewer) a desktop application for the analysis, visualization and data-mining of large-scale genomic data. We used false discovery rate, which defined the expected proportion of false positives among the declared significant results. FDR of 5% was used for the differential gene expression analysis of TP1 and TP2, while using significance analysis of microarray (SAM) in order to identify the significant genes for this group. The differential expressed genes were analysed as paired samples.

### Pathways enrichment analysis

We used the commercial software MetaCore™ (GeneGo, MI, USA). The differentially expressed genes from the MeV analysis were further analysed to identify the biological networks using the GeneGo Maps modules and GeneGo Folders of the program. Metacore™ conducts functional analysis in the form of network pathways based on a manually curated database of human protein–protein, protein–DNA and protein–compound interactions, metabolic and signalling pathways and the effects of bioactive molecules on gene expression. From each group we selected the most significant pathways. A p-Value is calculated for the common and unique groups. The results are ranked by the –log(p-Value). By default the most significant results for the common part is displayed [22].

To further verify these findings, we further carried out gene enrichment analysis that consisted of mapping gene IDs of the dataset onto gene IDs in entities of built-in functional ontologies represented in MetaCore by pathway maps and networks. The DE list provided by MeV, was uploaded in the website of MetaCore™ from Thomson Reuters Systems Biology Solutions in order to compare our samples and learn what genes are overrepresented in functionally relevant pathways. This analysis assessed the overall functional character of the sample set, providing a ranked representation of ontologies that are most saturated or “enriched” with the input data. Each GeneGo Process Network represents a comprehensive biological process with a specific functional theme.

### Quantitative real time PCR (q-RTPCR)

RT-qPCR was used to corroborate the relationship between expression trends between microarray and RT-qPCR. 14 genes were selected to be used in RT-qPCR due to their high observed-score and relevance to this study. 5ng of total RNA was reverse transcribed using oligo d(T) and Superscript III followed by RNase H treatment (Invitrogen Life Technologies), according manufacture’s protocol. PCR primers were designed for the genes selected on the basis of the microarray data as well as for the control genes (GAPDH: Glyceraldehyde 3-phosphate dehydrogenase) using Primer 3 (http://frodo.wi.mit.edu/primer3/). The cDNA was subjected to RT-PCR with defined primers and SYBR Green (Invitrogen Life Technologies) using MX3000p Stratagene real-time cycler (Stratagene, La Jolla, CA, USA). The data were analysed using the MxPro™ QPCR software version 4.0.1 (Stratagene, La Jolla, CA, USA). For all the experiments duplicates were used and relative mRNA expression was calculated by the comparative ΔΔCt method for all data. Values of fold changes represent averages from duplicates experiments. The data were further analysed by Wilcox test to check the statistical significance.

## Results

### Clinical profiles of patients enrolled in the study

The age range, CD4+ and CD8+ T cell counts, plasma viral load and HAART regimen that each patient received are shown in Table 1. All the patients enrolled in this study were used for blood collection at two time points, pre- and post-HAART. The separation between time points is shown in each case in Table 1. As it can also be seen from Table 1, all our patients responded to treatment post-HAART coinciding with the rise in CD4+ T cell counts. Thus, these patients were classed as responders, as they all achieved below-detectable plasma viremia post-HAART (<40 copies of HIV RNA/mL plasma).

**Table 1:** **Clinical profiles showing age, CD4+, CD8+ T cell counts, plasma viral load and the HAART regimen of patients enrolled in the study**

Patients	Age	Time point	CD4 count (cell/μl)	CD8 count (cell/μl)	Viral load (COPIES/ML)	Drugs used during treatment
SC18	27	20/08/96	510-pre	980	112,000	RTV
		2 years 07/09/98	710-post	840	< 20	SQV
						d4T
						3TC
SC19	36	day 3 19/09/96	610-pre	1408	108634	SQV
		3 years 08/09/99	720-pos	720	< 20	d4T
						3TC
SC 27	33	21/12/96	620-pre	1810	169841	SQV
		1.5 years 06/07/98	620-post	1200	< 20	d4T
						3TC
SC35	34	19/02/97	750-pre	1380	605236	RTV
		2 years 23/03/99	1010-post	840	< 20	SQV
						d4T
						3TC
SC39	43	04/04/97	n/a	n/a	1602	RTV
		3 years 05/04/00			< 20	SQV
						d4T
						3TC
SC40	34	SC peak 18/04/97	600-pre	400	3678	NVP
		on + off Rx 16/03/99	800-post	840	< 20	d4T
						3TC
SC51	35	pre 28/10/97	400-pre	680	10117	RTV
		1 year 28/10/98	810-post	1130	< 20	IDV
						d4T
						3TC
SC61	25	30/03/98	510-pre	930	6264	RTV
		2 years 11/05/00	990-post	870	< 20	IDV
						d4T
						3TC
SC65	26	20/05/98	360-pre	1380	647095	RTV
		2 years 10/05/00	620-post	1217	< 20	IDV
						d4T
						3TC
SC70	29	16/07/98	650-pre	1069	334088	RTV
		3 years 23/04/01	1222post	624	< 20	IDV
						d4T
						3TC
SC71	31	05/08/98	830-pre	1050	5208	RTV
		2 years 13/12/00	1073post	1073	<20	IDV
						d4T
						3TC
SC75	34	14/09/98	300-pre	1420	92000	RTV
		1 year 23/09/99	610-post	861	< 20	IDV
						d4T
						3TC
SC64	28	14/05/98	1050pre	950	24835	NEV
		2 years 21/03/00	1160post	1106	<20	ddI
						d4T
						3TC
DT 73	32	13/05/98	510-pre	831	142900	RTV
		3 year 26/09/00	722-post	399	< 20	IDV
						d4T
						3TC

Individual drug abbreviations of the prescribed anti-HIV drugs: 3TC (lamivudine); d4T (stavudine); ddI (didanosine); RTV (ritonavir); SQV (saquinavir); NVP (nevirapine); IDV (indinavir); NLF (nelfinavir); NEV (nevirapine). Patients stayed on treatment for 2-3 years in average.

Two time points were used, indicated as pre- and post-therapy in the genome-wide analysis.

### Hierarchical clustering analysis of the DE genes

We showed in Figure 1, the groups TP1 and TP2 separated into two clusters (TP1 = patients before treatment; TP2 = patients after treatment), clearly showing genomic distinction between TP1 and TP2 based on the differentially expressed gene list. This gave the confidence to the dataset to further derive specific DE list between TP1 and TP2 for downstream analysis.

### Differential expression analysis of genes between TP1 and TP2

The final list of DE genes for the comparison between TP1 (pre-therapy) and TP2 (post-therapy) contained 234 genes of which 212 DE genes were down-regulated and 22 up-regulated (see Additional file 1 for the DE genes list), suggesting significant separable differential expression between these two time points. This difference segregating the two biological entities between pre-and post-therapy time points, based on 234 DE genes, was considered confident enough for downstream pathway, gene networks and gene enrichment analysis.

**Figure 2:**
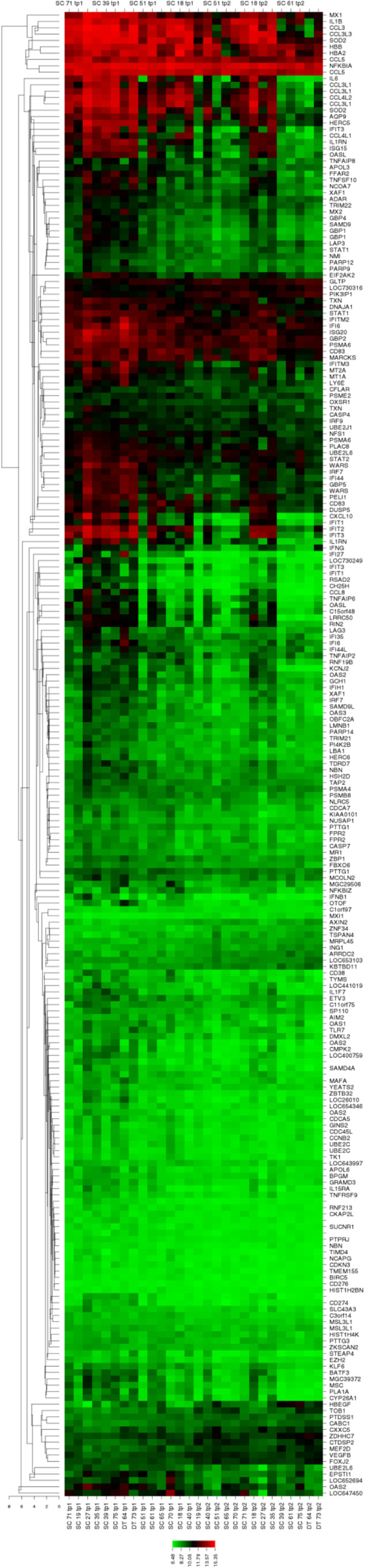
**Heatmap comparison of gene modulation between TP1 and TP2 groups.** This is a graphical representation of the data (234 DE genes), where the individual values contained in a matrix are represented as colors: **Red** means up-regulation and **Green** down-regulation.

Further, the MultiExperiment Viewer (MeV) was then used to address the differences in analysis requirements between this data type and traditional gene expression data. Its tools include automatic conversion functions from raw count data to processed values and differential expression detection and functional annotation enrichment detection based on published methods [17]. Using a website called CIMminer, which generates color-coded Clustered Image Maps (CIMs) (“heat maps”) to represent “high-dimensional” data sets such as gene expression profiles, we derived a heat map (Figure 2), which confirmed the differential expression of genes between two groups; showing strong segregation between TP1 and TP2 based on the DE genes.

### Pathway enrichment analysis

#### Pathway map prediction reveals dysregulation of intrinsic Immune response pathways during HIV-1 infection

Following a clear segregation between TP1 and TP2, next we analysed the functional importance of the DE gene list at the pathway level in order to derive functionally annotated genes relevant to TP1 and TP2. Genetic pathway map folders were evaluated using the MetaCore Analytical Suite (GeneGo Inc) (see Additional file 2 for legend of the pathways). The aim of this analysis was to determine what effect virus has on the immune system (seen in TP1) and how antiretroviral drugs ease the heightened gene expression, when virus is suppressed to below detectable levels with therapy in TP2 (see Additional file 1 for the genes involved in the pathways).

**Figure 3:**
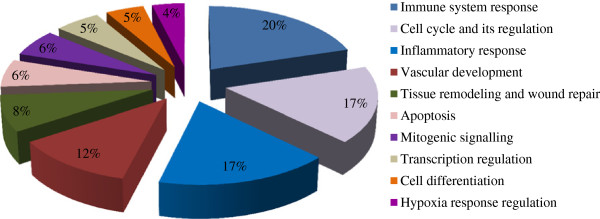
**Representation of the Top 10 GeneGo Pathway Maps from Metacore.** Significant pathways are represented in the form of a pie chart where each part represents -log_10_ of the P-value of that pathway from the set of over represented pathways, where the total of these -log_10_ *p value*s is 1. The *p value*s were determined by Metacore Pathway analysis based on the chi-squared value for the expected compared with observed number of genes identified from that pathway.

Initial analysis of the DE genes between TP1 and TP2 was performed in order to define the functional distribution of DE genes using MetaCore. Through these analyses, 32 pathway map folders were detected (see Additional file 3). Among the top 10 overrepresented and statistically significant pathway map folders belonged to Immune system response (20%) (*p value* 2.824e-7), followed by cell cycle (*p value* 3.224e-6), and Inflammatory response (*p value* 3.794e-6) (each 17%) Vascular development (12%) (*p value* 1.478e-4), Tissue remodelling (8%) (*p value* 1.882e-4), Apoptosis (*p value* 2.491e-3), mitogenic signalling (6%) (*p value* 1.015e-2) and Transcriptional regulation (5%) (*p value*1.137e-2), respectively (Figure 3).

**Figure 4:**
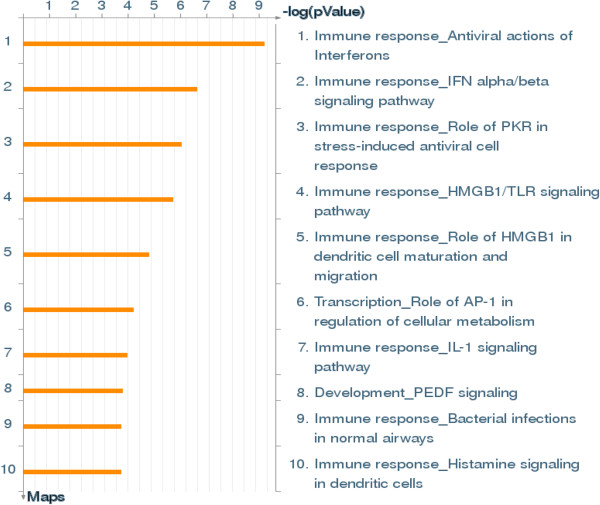
**Top 10 significantly enriched pathways derived through comparison of DE genes between time point 1 (TP1) and time-point 2 (TP2) of the same patients before and after treatment (FDR < 0.05).**

Further, we used the MetaCore package to analyze the differentially expressed genes included in Table 1. A 5% FDR (*p value* < 0.01) analysis produced 50 pathway maps (see Additional file 4 for the full list of pathway maps). Of these, the top 10 gene maps are summarized in Figure 4 and Table 2 and assembled according to and in the order of their significance. As seen in the Pie Chart distribution, the immune response system was the top ranked category, the pathway enrichment concurred with the map folders and we obtained 8 of top 10 significantly enriched pathways between TP1 and TP2 predominantly in the immune response category (Figure 4) supported by strong *p value*s (Table 2). This immune response category comprised of antiviral actions of Interferons and their signalling, stress-induced antiviral response, dendritic cell maturation and migration, TLR signalling and metabolism. Two additional maps that were also significant were Transcriptional Regulation of cellular metabolism and Development of PEDF signalling. It is apparent from the data shown in Table 3 and Figure 4 that of all immune responses, the involvement and up-regulation of interferon-related genes and genes related to inflammation predominated TP1-the time point before the initiation of HAART followed by their down-regulation following HAART.

**Table 2:** **MetaCore Map Folders and Pathways identified in the comparison between TP1 and TP2 (p < 0.01, FDR < 0.05)**

Map folder	^╟^p-value	†Ratio	Pathway maps	^╟^p-value	†Ratio
Immune system response	2.824e-7	21/1000	Immune response_IFN alpha/beta signaling pathway	8.104e-5	5\24
Cell cycle and its regulation	3.224e-6	14/516	Development_PEDF signaling	2.255e-2	6\53
Inflammatory response	3.794e-6	17/775	Immune response_HMGB1/TLR signaling pathway	6.734e-4	5\36
Vascular development (Angiogenesis)	1.478e-4	12/543	Development_PEDF signaling	1.689e-2	4\49
Tissue remodeling and wound repair	1.882e-4	12/557	Cell adhesion_Cell-matrix glycoconjugates	4.201e-2	3\38
Apoptosis	2.491e-3	14/953	Development_PEDF signaling	4.224e-3	4\49
Mitogenic signaling	1.015e-2	9/562	Development_PEDF signaling	4.656e-3	4\49
Transcription regulation	1.137e-2	3/71	Apoptosis and survival_Role of PKR in stress-induced apoptosis	4.099e-1	3\53
Cell differentiation	1.656e-2	12/940	Some pathways of EMT in cancer cells	2.340e-2	3\51
Hypoxia response regulation	3.269e-2	2\43	Development_EPO-induced PI3K/AKT pathway and Ca(2+) influx	1.000e + 0	2\43

^╟^p-value provided by MetaCore™ from GeneGo, Inc. Pathway analysis; † ratio meaning the number of differentially regulated pathway genes versus total number of genes comprising the pathway; IFN (Interferons); *PEDF,* (*Pigment epithelium-derived factor*); *HMGB1 (*High mobility group 1); PKR, (*Protein kinase R*);EMT (Epithelial to Mesenchymal Transition); EPO (Erythropoietin); PI3K, (Phosphoinositide 3-kinase); AKT, (Protein Kinase B); Ca (Calcium).

*p-values* and ratios of differentially expressed genes are shown.

**Table 3:** **Generation of biological networks by MetaCore™ for the defense response for the disease section of the enrichment analysis**

Key network objects	GO processes	p-Value
AKT(PKB), Caspase-9, HMGB1, Bcl-XL, IKK-beta	Regulation of defense response (74.0%), regulation of response to stress (80.0%), regulation of immune system process (82.0%), regulation of response to stimulus (92.0%), toll-like receptor 1 signaling pathway (40.0%)	1.31e-16
ATM, p21, MLL/MEN fusion protein, E1B-AP5, HEXIM1	Regulation of cell cycle (38.0%), cell cycle checkpoint (24.0%), chromatin organization (30.0%), negative regulation of cellular process (60.0%), chromatin modification (28.0%)	4.22e-39
CD19, CD79 complex, MHC class II, CD79B, IL-8	Immune response-regulating cell surface receptor signaling pathway (53.1%), positive regulation of T cell activation (55.1%), T cell co-stimulation (44.9%), lymphocyte co-stimulation (44.9%), response to interferon-gamma (49.0%)	1.82e-32
G3BP1 (hdhVIII), H-Ras, GPIAP1, ACSL3, IFNAR2	Leukocyte migration (46.0%), cell activation (54.0%), cell migration (52.0%), hemostasis (48.0%), localization of cell (52.0%)	2.05e-29
Casein kinase II, alpha chains, CD4, HSP27, IRF4, HDAC6	Regulation of macromolecule metabolic process (87.8%), regulation of primary metabolic process (87.8%), developmental process (87.8%), regulation of cellular metabolic process (87.8%), regulation of cellular biosynthetic process (79.6%)	1.40e-29

This is a variant of the shortest paths algorithm with main parameters of relative enrichment with the uploaded data and relative saturation of networks with canonical pathways. These networks are built on the fly and unique to the uploaded data. In this workflow the networks were prioritized based on the number of fragments of canonical pathways on the network.

#### Scoring and prioritization of networks/pathways according to the relevance to input data

**Figure 5:**
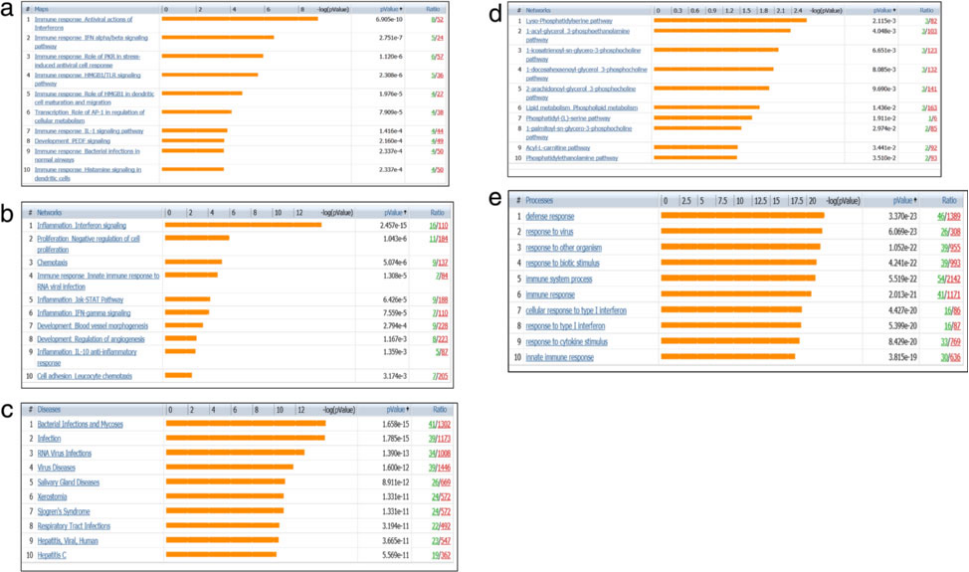
**Enrichment in functional ontologies for the comparison between patients before and after treatment.** Enrichment analysis across five ontologies in MetaCore: **(a)** GeneGo pathway maps; **(b)** GeneGo networks; **(c)** GeneGo disease by biomarkers **(d)** GO processes and **(e)** GeneGo processes. The associated false discovery rate is less than 0.01 for a p-value threshold less than or equal to 0.01.

Next, we analysed the DE genes in MetaCore networks in order to address the issue of how different networks and pathway modules in MetaCore can be prioritized based on their statistical significance (Figure 5a-e) with respect to our experimental datasets. Significance was evaluated based on the size of the intersection between user’s dataset and set of genes/proteins corresponding to a network module/pathway in question.

**Figure 6:**
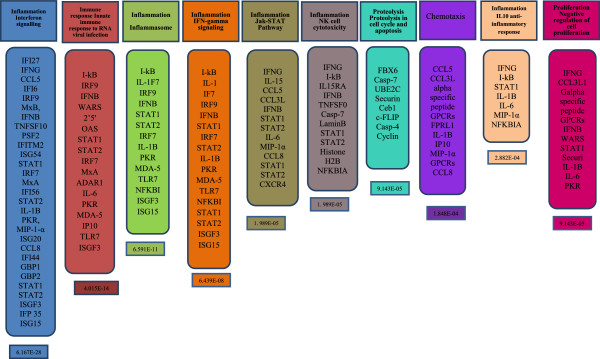
**Process Networks by Enrichment analysis in MetaCore™ software; these are the top 10 process network pathways accordingly with their p-value calculated by the program.**

The resulting networks, if significant, will mean that the algorithm have succeeded in creating modules that have higher than random saturation with the genes of interest, which was the case with our dataset shown in Figure 5(b) under process networks. As can be seen in Figure 6, that of the top 10 process networks, 5 belonged to inflammation (all enriched in interferon signalling) and immune response to virus infection, whereas other 5 were in the development, cell adhesion and cell-proliferation. Thus, the top ranked network was again in inflammation of Interferon signalling, which again concurs with the pathway analysis shown in Figure 4.

Further, the examination of disease by biomarker folder was also consistent with top 3 maps related to RNA virus infection, which was again consistent with the GO processes (Figure 5e) with the top 2 maps in the defence response and response to virus. Thus, the process networks, disease by biomarkers and GO processes were all in concordance with the DE genes relevant to immune response to viral infection and RNA virus, which are highly relevant to HIV infection.

**Figure 7:**
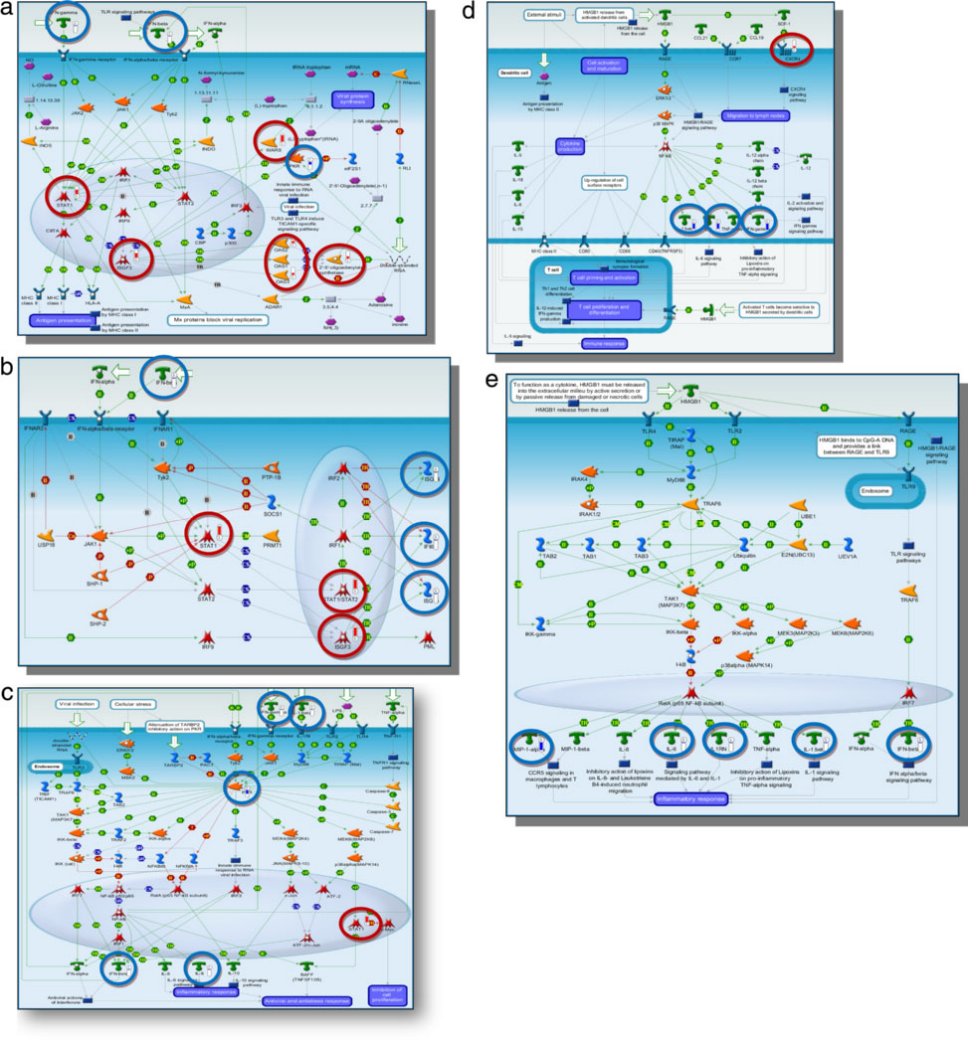
**Canonical pathway maps represent a set of about 650 signalling and metabolic maps covering human biology (signalling and metabolism) in a comprehensive way.** All maps are drawn from scratch by GeneGo annotators and manually curated and edited. Experimental data is visualized on the maps as blue (for downregulation) and red (upregulation) histograms. The height of the histogram corresponds to the relative expression value for a particular gene/protein (MetaCore™). **a)** Immune response_Antiviral actions of Interferons pathway; **b)** Immune response_IFN alpha/beta signaling pathway; **c)** Immune response_Role of PKR in stress induced antiviral cell response; **d)** Immune response_HMGB1/TLR signaling pathway; **e)** Immune response_Role of HMGB1 in dendritic cell maturation and migration.

The Figure 6 shows the ten most enriched GeneGo processes for the sample set as by their *p value*. 8 of 10 enrichments, which fully represent only the DE genes between TP1 and TP2, were in the inflammation/signalling and immune response. A number of these DE genes are overlapping between the networks as most of them are involved in the immune system response and inflammation. The representation of all significant genes in the top ranked 5 pathways is shown in Figure 7a-e.

Another significant and the most notable feature in our analyses was the predominance and up-regulation of genes related to immune response and inflammation in TP1, which was also concordant with the map folders, process networks and GO processes (Figure 5). The visualization of GO process network shown in Table 4 showed that the functional annotation of our DE genes in defense response (the top ranked category), comprised of genes related to interferon, interferon-induced or interferon-associated (involved in innate immune response, defense response, regulation of innate immune and defense response, surface receptor signalling, cytokine-mediated pathways, etc) and were highly up-regulated in TP1 when compared to TP2 supported by strong statistical support (Table 3). In these top 5 pathways, 28 of our DE genes (Table 4) were related to interferons, antiviral function, interferon signalling, interferon-induced genes and immune-response/activation genes with high statistical significance. Thus, the immune and inflammatory systems with possible involvement of the interferon (IFN) family and also the STAT (signal transducers and activators of transcription) family were all significant in this comparison (Table 3).

**Table 4:** **Over representation of genes significant in TP1 versus TP2 comparison, based on the top 5 significant pathways**

Gene annotation	***p-value***	Cell function	Regulation
IFI6	p < = 0.001198	Regulation of apoptosis	Down
IFN- β*	p < = 0.001647	Antiviral activity which is Mainly involved in innate immune response	Down
IFN-γ*	p < = 0.008242	Critical for innate and adaptive immunity against Viral and intracellular bacterial infections and for tumor control	Down
IL-1β*	p < = 0.005439	An important mediator of the inflammatory response, and is involved in a variety of cellular activities, including cell proliferation, differentiation, and apoptosis	Down
IL-6*	p < = 0.002616	Acts as both a pro-inflammatory and anti-inflammatory cytokine; stimulate immune response	Down
IL1RN	p < = 0.0007325	Inhibits the activity of IL-1 by binding to its receptor	Down
ISG15	p < = 0.0007325	Antiviral activity during viral infections	Down
ISG54	p < = 0.0004398	Restrict virus infection through alteration of cellular protein synthesis	Down
MIP-α	p < = 0.0003696	Involved in the acute inflammatory state in the recruitment and activation of polymorphonuclear leukocytes	Down
OAS1*	p < = 0.002616	Involved in the innate Immune response to viral infection	Up
OAS2*	p < = 0.004717	Involved in the innate Immune response to viral infection	Up
OAS3*	p < = 2.622e-05	Involved in the innate Immune response to viral infection	Up
PKR*	p < = 0.01396	Inhibits further cellular mRNA translation, thereby preventing viral Protein synthesis; induce cellular apoptosis, to prevent further viral spread	Down
STAT1	p < = 0.002616	Signal transducer and activator of transcription that mediates signaling by interferons	Up
STAT2	p < = 0.01797	Signal transducer and activator of transcription that mediates signaling by type I IFNs	Up
WARS	p < = 0.006259	Catalyzes the aminoacylation of tRNA(trp) with tryptophan and is induced by interferon; associated with angiogenesis	Up

*Genes that were validated by RT-PCR in this study: IFI6, (Interferon alpha-inducible protein 6); IFN- β, (Interferon beta); IFN-γ (interferon gamma); IL-1β (Interleukin-1 beta); IL-6, (interleukin 6); IL1RN, (interleukin 1 receptor antagonist); ISG, (interferon stimulated gene); MIP, (Major intrinsic protein of lens fiber); OAS (2′-5′-oligoadenylate synthetase); PKR, (protein kinase R); WARS, (Tryptophanyl-tRNA synthetase).

The 16 genes shown in the table are from the DE list and were found to be involved across 5 pathways, which were also validated by q-RT PCR.

Thus, these analyses unambiguously confirm the involvement and up-regulation of genes involved in the immune response and inflammation being the most significant driving force during the viremic (TP1) phase, which could be largely attributed to immune activation during viremic state (TP1). Most notable was the regulation in TP2, characterized by systematic down-modulation of the very genes, which predominated TP1. This clearly demonstrates that HIV not only plays a significant role in subverting the host gene machinery, but the gene expression is possibly the main driver of immune activation, which became evident from the reduction in plasma viremia to below detectable levels (<40 copies/ml) upon the initiation of HAART treatment, leading to down-modulation of the very genes that underlie viremia and immune activation.

### Quantitative real time PCR corroboration of 14 DE genes

**Figure 8:**
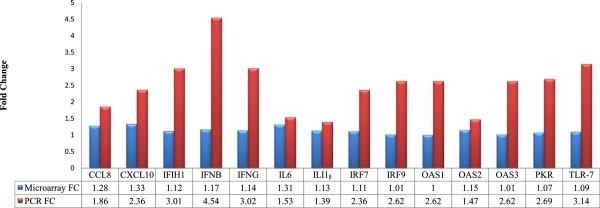
**qRT-PCR validation of 14 significant DE genes.** Correlation of the fold change of the 14 selected genes between qRT-PCR and microarray. Blue colour represents microarray fold change and the red colour represents qRT-PCR fold change. Individual fold changes values for qRT-PCR and microarray across all 14 genes are shown underneath each gene.

Following the microarray analysis, quantitative real-time PCR (qRT-PCR) was performed using the same RNA samples in order to functionally validate expression patterns of some of the functionally, but statistically significant genes between microarray and qRT-PCR. We analysed 14 such DE genes (CCL8, CXCL10, IFIH1, IFNB, IFNG, IL6, IL1Beta, IRF7, IRF9, OAS1 to 3, PKR and TLR-7) shown in Figure 8, which were directly or indirectly related to interferon function, signalling and immune response. All 14 DE genes showed consistent trends between qRT-PCR and microarray, implying their functional relevance (Figure 8).

**Figure 9:**
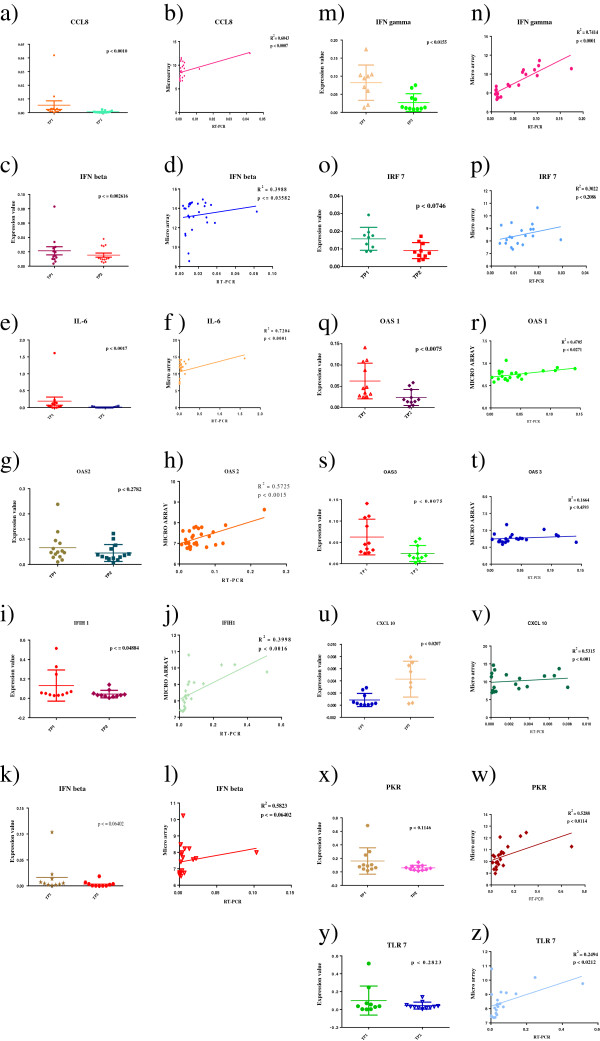
**Association of gene expression by real-time PCR of the DE found significant in our analysis.** Representative scatterplots with linear regression analysis confirm the association between expression levels between microarray and q-RTPCR **(figures b, d, f, h, j, l, n, p, r, t, v, y and z)** p*-values* for the expression between the different group were measured using the GraphPad Prism®software and also correlation values **(a, c, e, g, i, k, m, o, q, s, u, x and w)**. The significant ones were in the range of p < 0.001 and with the R^2^ value closer to 1.

With the help of GraphPad Prism® a more in-depth correlation analysis was performed for individual genes to validate the statistical significance of their expression in RT-qPCR followed by validation of correlation between microarray and RT-qPCR expression trends (Figure 9). For each of the genes, standard coefficient correlation and paired t test were performed to show how the genes expressed between different groups and also their significance. We identified 13 of 14 genes showing statistically significant relationship in expression trends between microarray and RT-qPCR, with OAS3 being the only exception (Figure 3). Although 13 of 14 genes showed confident R^2^ values, the genes PKR, CXCL10, CCL8, IL-6, OAS2, IFNbeta and IFN delta showing R^2^ value >0.5 coupled with excellent *p value*s (Figure 8). Although in RT-qPCR, only 11 of 14 achieved statistical significance, the three exceptions (PKR, TLR7 and OAS2) (Figure 9) showed perfect correlation in expression trends between microarray and RT-qPCR.

## Discussion

Even though there have been many reports of gene expression changes at different stages of HIV infection [23] the information on how genomic modulation occurs in HIV-infected individuals assessing whole transcriptome before and after HAART therapy and the genomic basis of the effects of anti-HIV drugs leading to recovery of genes involved in immune reconstitution is still very limited [15, 16, 24].

In this study, we have carried out a genome-wide transcriptomic analysis of primary PBMCs derived from 14 HIV + patients before and after the initiation of HAART therapy to ascertain whether HIV leads to perturbations in immunity-related genes and if so, how these immune function-related genes recover from insults incurred by HIV following HAART when virus becomes below detectable to <20 copies of HIV RNA/ml. These study patients were classed as responders, which was consistent with concomitant rise in their CD4+ T cell counts coupled with reduction in plasma viremia following HAART. The immune reconstitution fails under the value of 200 CD4+ T cells/ml, which is considered as a critical threshold and this occurs in 5–27% of patients receiving HAART [14, 25]. In fact, CD4 + T-cell counts persistently < 250 cells/ml or a percentage of CD4 T cells < 17, have been considered a sign of poor immune reconstitution [26]. In our study, all our patients showed CD4+ T cell counts of 300/uL blood and above. Therefore, we believe that successful HAART treatment in responders will better reflect the genomic basis of immunologic reconstitution and the genes involved in the immune system recovery.

We analysed the peripheral blood mononuclear cells in order to obtain a global snapshot of the differences in gene expressions emanating as a consequence of host-virus interaction. We believe that a single cell type will not be informative in the context of global gene expression. 234 differentially expressed genes were detected between pre-and post-therapy time points, which demonstrate the evidence for distinct transcriptional signatures that segregated the pre-therapy (viremic), and post-therapy (aviremic) time points of HIV + patients. The untreated stage (TP1) was characterized by up-regulation of genes that were related to immune system function, inflammation and activation with 8 of top 10 pathway maps functionally related to immune response genes encompassing antiviral interferons and their signalling, DC maturation and migration, IL-1 signalling, etc. Other 2 pathway maps were related to development and transcriptional regulation. In contrast, treated stage (TP2) when below detectable levels of plasma viremia (<20 copies/ml plasma) was achieved with successful HAART, systematic down-regulation of these functional categories was observed, suggesting that most of the alterations in gene expression observed in untreated stage resumed desired functional levels upon the initiation of HAART treatment. This finding is consistent with the work of Massanella et al. [24] and Li et al. [15], where they showed an abatement of innate immune response following treatment. It is important to iterate that post-therapy down-regulation of immune function related genes, specifically to interferon antiviral/signalling genes, inflammatory and cellular activation may be indicative of reduction in cellular activation which gradually occurs with potent antiretroviral therapy and more rapidly in responders- as evidenced here in this study. This argument becomes even more conceivable when the degree of T cell activation is compared between two groups. It has been shown that the degree of T cell activation is lower in controllers than in non-controllers, but higher than observed in HIV uninfected individuals and higher than observed in antiretroviral-treated patients [27]. This corroborates with our observations showing considerably reduced cellular activation levels upon successful HAART signalled by systematic down regulation of the very genes involved in these functional pathways. Therefore, it is plausible to hypothesize that gene expression and its modulation pre-therapy is possibly guided, in part, by cellular activation as a consequence of HIV infection, whereas the down-regulation of these functional pathways post-HAART aid immune reconstitution, pointing at possible genomic basis of immune-reconstitution upon successful HAART. Overall, this analysis showed an aspect of host gene expression pre-and post HAART and is consistent with the findings on the role of HAART treatment in slowing the rate of HIV disease progression and AIDS with improved patient outcomes [28].

The interferon (IFN) system is a well-studied branch of the innate immune system active against viruses. The infection of cells by many viruses provokes synthesis and secretion of IFNs, which mediates induction of a cellular antiviral state that obstructs further viral spread. For HIV, previous studies have implicated IFNs in blocking both early and late stages of the HIV-1 life cycle [29, 30]. Our data not only confirmed these previous studies, but also are unique in showing molecular signatures, up-regulation and over-representation of immune response genes enriched in IFN and IFN-related pathways with over 25 statistically significant genes during untreated HIV infection as opposed to the treated stage. A closer examination of top 5 pathways immune response pathways showed that even though considerable numbers of genes were overlapping between them, they were all functionally related to IFN family within the immune response folder. The top-ranking pathway was antiviral action of Interferon, enrichment of over 12 DE genes (IFN-beta, IFN-gamma, STAT1, STAT2, IRF9, WARS, PKR, OAS1, OAS2, OAS3, MxA, ADAR1) during untreated stage. All these genes have in common is the activation of the IFN family. It is known that HIV-1 interacts through multiple signalling pathways to reprogram the transcriptome and the proteome of host cells by subverting the host immune defense and antiviral function [31].

The second most significant pathway involved during the untreated stage was related to signalling of IFN-beta and gamma, both produced by immune cells. These interferons were also related to antiviral responses and are implicated in blocking both early and late stages of the HIV-1 life cycle [32]. Most of the genes overlapped with the top-ranked pathway and all of these genes were related to IFN signalling and they were all up-regulated during the untreated stages of HIV infection.

The third most significant pathway that we show here was related to HMGB1 and TLR signalling down-regulated in TP2 (Figure 7d). High mobility group box 1 (HMGB1) is a DNA-binding nuclear protein that can act as an alarmin, a danger signal to alert the innate immune system for the initiation of host defence [33]. They recognize distinct pathogen-associated molecular patterns and play a critical role in innate immune responses [34–36]. The most significant genes in this particular pathway were Ik-β, MIP1-α, IL-6, IL1RN, IL-1_β_, IRF7, IFN-β. Previous studies have shown that Toll-like receptor (TLR) pathway contributes to the persistent immune activation observed in chronically HIV-1–infected individuals. Even though the link between immune activation and HIV-1 disease progression is well established, the underlying genomic basis through which HIV-1 induces immune activation remains unclear. In our study, the TLR7 was enriched in the top ranked immune response pathway (p < 0.001926). It has been demonstrated that HIV-1 encodes for multiple TLR7/8 ligands that can mediate direct activation of the immune system *in vitro*[37]. Moreover, evidence for a role of chronic TLR stimulation in HIV-1 pathogenesis has been recently described in a murine model, which showed that chronic activation of TLR7 can directly lead to immune activation as well as an array of immune dysfunction similar to those observed in human HIV-1 chronic infection [38]. Interestingly, recent data from the non-human primate model of HIV-1 infection provided additional support for a role of the TLR7 pathway in HIV-1 pathogenesis [39]. Apart from this, other genes involved in immune activation (such as CD38, CD83 and TNFSR family) were also significantly expressed and were up-regulated during the untreated phase, suggesting immune activation as one key aspect of the viremic stage and acute infection, which is also consistent with the increased gene expression changes associated with increased plasma viral loads in viremic patients [10].

The most notable aspect of these studies is an unambiguous demonstration of up-regulation of the most of the pathways related to immune response during the untreated stage of HIV infection followed by down-regulation of the same pathways observed during the HAART-treated stage of HIV infection. By up-regulating most of the pathways related to the IFN system, the host cells could be more in control of virus replication which will determine the course of the infection. It becomes more conceivable, when we see that the increase of IFN production is negatively linked to viral loads. Therefore, it is obvious that the immune activation in patients without HAART treatment affected by the virus is trying to protect the disease spread, which is a beneficial aspect aiding the untreated stage. If we compare this scenario in chronically infected HIV patients, who control virus in the absence of therapy and have below detectable HIV, there is an emerging consensus that much of the inflammatory response related to cellular activation over time does more harm than good.

What we have shown here is that the virus causes more profound functional changes by subverting and in manipulating the host cell gene machinery by regulating the most important part of the host immune system, thereby leading to inflammation and immune system activation. In this context, not only the IFN system pathway and the immune response pathways were the most significant, but the high mobility group box 1 (HMB1) was also up-regulated in patients before treatment, which signals heightened innate immune responses to virus infection. This pathway has more profound importance and is related to the response to exogenous pathogens molecules, acting as a danger signal and triggering inflammation [28]. That means that the HIV-1 virus regulates all the levels of the innate immune response including the signalling inflammation by up-regulating the genes related to this role such as STAT-1, MIP-1α, IL-6, IL1RN and IL1β, as seen in our study. The affirmation to this proof is reflected in the down-modulation of these very genes upon successful HAART and below detectable levels of plasma viremia (TP2) in our study.

Overall, our study is unique not only in demonstrating how virus subverts the host gene machinery by up-regulating key immune response and genes involved in inflammation and cellular activation, but also in showing that HAART therapy modulates gene expression and overcomes the insults that are neutralized upon the initiation of HAART therapy. In simple terms, the successful outcome of HAART therapy can now be visualized genomically through the down-regulation of particular immune and inflammation genes, as this study has shown. It is known that therapy leads to the reconstitution of the immune system and we show that the down-regulation of the most important genes related to immune system response and inflammation may provide respite from cellular activation/inflammation in HIV patients. HAART treatment can improve clinical, virologic, and immunologic characteristics, but very little is known of the host molecular mechanisms underlying untreated versus HAART treated stage of the same patient. Our work shows that although there is a common set of key genes associated with HIV with altered expression in both stages, each stage (pre-and-post therapy) was characterized by unique molecular signatures, which have immense clinical significance.

In summary, the results presented in this study offer new comparative insights related to disease status that can distinguish differentiated patterns of gene expression between HIV patients before and after HAART, which can reveal how genomic insults incurred by HIV recover following the initiation of HAART. Taken together, the innovative approach we have used can be applied to disease conditions other than HIV where correlating complex cellular and molecular signatures of immunity may provide new insights in the mechanisms of pathogenesis and success of therapy.

## Conclusion

Our results demonstrate for the first time a clear genome-wide distinction between HIV patients pre-and post highly active antiretroviral therapy. They clearly show that immune activation and inflammation play a significant role in host gene expression in HIV + individuals with viremia. Affirmation to these observations came from successful HAART therapy down-regulating the very genes and pathways that guided viremia and immune activation. This is the first study to show key genomic differences pre-and post-HAART in the same patient, highlighting how virus subverts the host gene machinery and how antiretroviral drugs neutralize this effect at the level of host genome, thereby providing the first snapshot of genomic basis of immune deterioration during viremia and its reconstitution upon successful HAART. Many of the genes and pathways identified in this study will further facilitate the development of new generation of diagnostic markers and novel genome-based strategies for therapeutic interventions for HIV patients.

## Electronic supplementary material

Additional file 1: **Differentially expressed gene list derived through comparison of genes between TP1 versus TP2 based on their observed scores.** A total of 234 genes were differentially expressed between TP1 and TP2. (PDF 324 KB)

Additional file 2: MetaCore™ pathway legend pictures. (PDF 185 KB)

Additional file 3: **32 map folders corresponding from the comparison between TP1 and TP2.** (PDF 180 KB)

Additional file 4: **Pathway maps of the comparisons between TP1 versus TP2.** (PDF 132 KB)
