# Supplementary material for: Genome-wide analysis of primary peripheral blood mononuclear cells from HIV + patients-pre-and post- HAART show immune activation and inflammation the main drivers of host gene expression
Source: Mol Cell Ther. 2014 Apr 3;2:11. doi: 10.1186/2052-8426-2-11 (PMC4451969; doi:10.1186/2052-8426-2-11)
Supplement: Supplementary file 1 — Additional file 1: Differentially expressed gene list derived through comparison of genes between TP1 versus TP2 based on their observed scores. A total of 234 genes were differentially expressed between TP1 and TP2. (PDF 324 KB) [file 40591_2013_14_MOESM1_ESM.pdf]

Additional file 2. Differentially expressed gene list derived through comparison of genes between TP1 versus TP2 based on their observed scores. A total of 234 genes were differentially expressed between TP1 and TP2.

Gene symbol	Expected score	Observed score	Numerator	Denominator	Fold change
**OAS3**	0.42675933	-5.0803823	-1.2797203	0.25189447	0.3734844
**FFAR2**	-0.5644532	-4.9500237	-1.9625149	0.39646575	0.30037153
**CCL4L2**	-0.9398435	-4.9085317	-3.186326	0.64914036	0.23708229
**GBP1**	-0.504601	-4.6164107	-1.6606779	0.35973358	0.27789873
**CCL3L1**	-0.9405141	-4.614468	-2.8382845	0.61508375	0.2062823
**CCL3L1**	-0.9408498	-4.534406	-2.800703	0.617656	0.26621494
**CCL3L1**	-0.9406826	-4.519408	-2.86477	0.6338816	0.2999001
**IFIT2**	-0.06419801	-4.4181805	-2.7349148	0.61901385	0.25621757
**CCL3**	-0.94101036	-4.3225007	-2.7621813	0.6390239	0.31335104
**C3orf14**	-1.0526695	-4.25157	-0.4228077	0.09944742	0.74170494
**IL1RN**	-0.0532208	-4.2384005	-2.423664	0.5718346	0.19622545
**IFI27**	-0.065098606	-4.19814	-1.9500985	0.46451488	0.11236938
**SAMD9L**	0.7309575	-4.180682	-1.3197756	0.31568426	0.365504
**IFIT3**	-0.06411307	-4.1685696	-3.0679684	0.7359763	0.15986454
**IFI44L**	-0.06476419	-4.127145	-1.2851315	0.31138512	0.39291248
**GBP1**	-0.50449896	-4.1266117	-1.6439705	0.39838263	0.26559356
**EPSTI1**	-0.62839705	-4.0719585	-1.2264242	0.30118778	0.40335783
**SAMD9**	0.73082536	-4.05626	-1.3938246	0.34362307	0.3717935
**CXCL10**	-0.76003265	-4.021757	-3.1006546	0.7709701	0.1341035
**IFIT3**	-0.06395177	-4.0161567	-1.6345525	0.4069942	0.28886032
**GBP5**	-0.50410503	-3.9744053	-1.4250298	0.35855168	0.3122645
**IFIT1**	-0.06444321	-3.9705477	-2.4483986	0.61664003	0.25112563
**ETV3**	-0.6194654	-3.9242573	-.70651674	0.18003833	0.5836812
**PLA1A**	0.5115807	-3.8697472	-1.1976147	0.30948138	0.4194429
**IFIT3**	-0.06402939	-3.8242953	-3.0442371	0.79602563	0.2485131
**MSC**	0.34185186	-3.8098137	-1.1936555	0.3133107	0.41341317
**ISG15**	-0.038145613	-3.7631938	-2.3269339	0.61834013	0.31049064

**CCL8**	-0.93902236	-3.754046	-2.102775	0.56013566	0.18482934
**CCL4L1**	-0.94002074	-3.741667	-2.386836	0.63790715	0.31420437
**LBA1**	0.02064815	-3.7316766	-0.73635674	0.19732597	0.5507719
**CCL5**	-0.93951505	-3.7280154	-0.77837944	0.20879191	0.58271605
**CCL3L3**	-0.9403485	-3.7213936	-2.126958	0.57154876	0.41006738
**IL1RN**	-0.05313881	-3.7021108	-2.2973814	0.6205599	0.26846543
**KCNJ2**	-0.024531571	-3.696016	-1.4922967	0.40375817	0.44253558
**XAF1**	1.487174	-3.6725726	-1.4011335	0.38151282	0.4016438
**SOD2**	0.8849008	-3.6665595	-1.7177505	0.4684911	0.4575416
**MT2A**	0.34545022	-3.66402	-1.445199	0.3944299	0.36470655
**KIAA0101**	-0.018818974	-3.6500738	-0.81513834	0.22332107	0.5250399
**CD38**	-0.91713107	-3.645084	-0.8706393	0.23885302	0.47886088
**IFI6**	-0.06468714	-3.6411843	-1.3402395	0.3680779	0.30766064
**CD83**	-0.9091399	-3.6375957	-1.1473904	0.3154255	0.4749613
**TLR7**	1.0585091	-3.6269853	-0.8278775	0.228255	0.53850114
**IFNB1**	-0.06270272	-3.6226375	-1.0422235	0.2876974	0.39908975
**CDCA7**	-0.89668834	-3.6170485	-0.73892546	0.20428962	0.58981645
**PARP14**	0.4595197	-3.599667	-0.7932458	0.22036643	0.55762637
**SOD2**	0.8847393	-3.5959492	-2.2798367	0.6340014	0.37602705
**RNF19B**	0.67937505	-3.5890775	-1.1760731	0.32768115	0.48817736
**GCH1**	-0.5029993	-3.5863428	-1.0650482	0.29697335	0.4695963
**IFI6**	-0.0646093	-3.5668287	-1.0072136	0.2823835	0.42085356
**GBP2**	-0.5044018	-3.5198333	-0.8450451	0.240081	0.5294051
**ZBP1**	1.5417188	-3.512325	-0.58825207	0.16748224	0.6483138
**OAS1**	0.42598832	-3.4865394	-0.752604	0.21585989	0.58638155
**IL6**	-0.050994974	-3.4754646	-2.9971752	0.86238116	0.29427785
**LY6E**	0.26441905	-3.4647062	-0.9971714	0.28780836	0.4338826
**UBE2C**	1.2625195	-3.4587138	-0.46288824	0.13383248	0.699815
**HSH2D**	-0.075736806	-3.4559553	-0.8123541	0.2350592	0.52780247
**MT1A**	0.34473285	-3.4507453	-1.1660624	0.33791608	0.44624764
**PSMA6**	0.5800359	-3.4336317	-0.57785034	0.1682913	0.6502408
**GBP4**	-0.50420433	-3.4330473	-1.2520523	0.3647058	0.36935398

**IFI44**	-0.06484902	-3.4326754	-1.4650555	0.42679697	0.3603535
**LOC730249**	0.24238099	-3.4314592	-1.8182669	0.52988154	0.14167856
**CD83**	-0.90929866	-3.4306986	-1.0062847	0.29331774	0.5584771
**FPR2**	-0.5278663	-3.43036	-0.68151426	0.19867136	0.6079879
**IFITM3**	-0.063616134	-3.418472	-1.4453506	0.42280605	0.33314458
**NUSAP1**	0.42417964	-3.4179163	-0.49759436	0.14558412	0.68396235
**STAT1**	0.9362078	-3.4050975	-0.99912643	0.2934208	0.5411204
**LOC400759**	0.07213392	-3.3992145	-0.88064146	0.25907204	0.4837633
**TYMS**	1.2502244	-3.3480518	-0.6773577	0.20231397	0.5908364
**PSMA6**	0.5801418	-3.342626	-0.78498745	0.23484154	0.58253014
**LRRC50**	0.25834143	-3.3350925	-2.088745	0.626293	0.38605222
**KLF6**	0.003711841	-3.3349445	-0.6727071	0.20171462	0.63519365
**PTTG1**	0.60013187	-3.3137422	-0.49916553	0.15063499	0.69452864
**HBB**	-0.44289204	-3.312816	-1.2503223	0.3774198	0.48730302
**IL1B**	-0.054126855	-3.309459	-1.7789259	0.5375277	0.48973417
**PTPRJ**	0.5985735	-3.3067906	-0.2056756	0.062197953	0.8639993
**LOC654346**	0.22850724	-3.3059611	-0.40951014	0.123870224	0.73209304
**FBXO6**	-0.5733568	-3.29039	-0.46081352	0.1400483	0.721025
**NFS1**	0.3875634	-3.259498	-0.656291	0.20134728	0.6669095
**PARP12**	0.45942268	-3.2430842	-0.8509655	0.2623939	0.4851226
**OAS2**	0.42656448	-3.2389882	-1.341341	0.41412348	0.4083785
**PARP9**	0.4607717	-3.230047	-0.80021	0.24773943	0.53216743
**PTTG1**	0.60002154	-3.2259624	-0.47765923	0.1480672	0.71703345
**OTOF**	0.44560736	-3.224663	-0.91716146	0.28442088	0.40973353
**APOL3**	-1.488796	-3.2170537	-0.9260073	0.2878433	0.50424623
**TMEM155**	1.0786825	-3.215469	-0.20744467	0.06451459	0.8626798
**TNFSF10**	1.1214488	-3.2112827	-1.6090012	0.50104624	0.31774375
**ADAR**	-1.8603022	-3.1969717	-0.72576237	0.22701558	0.59331834
**FPR2**	-0.52797174	-3.1962204	-0.6374574	0.19944099	0.6590889
**TIMD4**	1.0469642	-3.18891	-0.32158947	0.1008462	0.78602266
**ZBTB32**	1.5491271	-3.1869264	-0.62415266	0.19584784	0.5841406
**TNFAIP8**	1.1154293	-3.1658072	-0.95710564	0.30232593	0.4711884

**PSMA4**	0.579815	-3.1372805	-0.4705782	0.14999558	0.7016273
**CDCA5**	-0.8968436	-3.1360571	-0.51769686	0.1650789	0.6618505
**OAS2**	0.42646512	-3.135491	-0.8076601	0.25758648	0.513324
**RSAD2**	0.7152234	-3.128006	-1.2728581	0.40692317	0.3136008
**TAP2**	0.9856252	-3.1249948	-0.59969234	0.19190188	0.6277214
**XAF1**	1.4876466	-3.1228774	-0.99207497	0.31767976	0.46484175
**IFIH1**	-0.064525485	-3.121719	-1.0734649	0.3438698	0.40213183
**NBN**	0.3686791	-3.1144345	-0.7322626	0.23511897	0.5582653
**LAP3**	0.01751892	-3.1143086	-0.91018105	0.29225782	0.49493554
**OASL**	0.42694467	-3.106628	-1.9805574	0.63752645	0.34732276
**LAG3**	0.015447804	-3.098014	-1.179234	0.3806419	0.31881177
**BIRC5**	-1.2440537	-3.0928712	-0.19627857	0.06346161	0.872191
**STEAP4**	0.939546	-3.0919583	-0.60287476	0.19498153	0.6420472
**MAFA**	0.2701851	-3.087104	-0.5638318	0.18264101	0.64719933
**HBA2**	-0.44298798	-3.086008	-1.4379082	0.4659444	0.4566744
**TRIM22**	1.1695155	-3.0740912	-0.7305336	0.23764214	0.5921833
**OAS2**	0.42627576	-3.070014	-0.47762632	0.1555779	0.68688357
**ISG20**	-0.038061373	-3.0673218	-1.018199	0.3319505	0.45157266
**OAS2**	0.4263685	-3.0623052	-1.2564921	0.41030926	0.4620832
**UBE2L6**	1.2725362	-3.0616794	-.64707184	0.21134539	0.66142195
**TXN**	1.2415017	-3.053	-.75183487	0.24626102	0.61039346
**WARS**	1.4099236	-3.0514135	-1.2479172	0.40896365	0.30912063
**TNFAIP6**	1.1149546	-3.048315	-1.571579	0.51555663	0.31398633
**IRF9**	-0.0394065	-3.0399346	-.52831554	0.17379175	0.6866199
**TXN**	1.2417754	-3.039867	-.73644066	0.24226083	0.59636915
**NCOA7**	0.37426957	-3.032179	-1.0827312	0.35708025	0.47810298
**MARCKS**	0.28404266	-3.0305226	-0.9809265	0.3236823	0.5996619
**RNF213**	0.6796081	-3.0290391	-.28264952	0.09331326	0.81055146
**IRF7**	-0.0395729	-3.0225742	-0.9853792	0.32600662	0.46420413
**TDRD7**	1.0171225	-3.0168214	-0.8000927	0.2652105	0.5338549
**C1orf97**	-1.0846637	-3.0165353	-.22026396	0.07301886	0.8527123
**NFKBIZ**	0.38729304	-3.0157874	-0.7810879	0.25899965	0.51715183

**STAT1**	0.93603957	-3.0129788	-.90178394	0.2992998	0.5234202
**MX2**	0.3543741	-3.0050066	-1.0762224	0.35814312	0.4199904
**NA**	-0.27473208	-2.995216	-0.5131979	0.1713392	0.693166
**MX1**	0.35428262	-2.994807	-1.3634386	0.4552676	0.37525997
**PSME2**	0.58569604	-2.9932623	-0.5481138	0.18311587	0.7043533
**IFI35**	-0.06493276	-2.9897797	-1.0024805	0.33530247	0.41527668
**CKAP2L**	-0.84843785	-2.9789152	-0.26414204	0.088670544	0.82421356
**IFITM2**	-0.06370018	-2.9764738	-0.80049706	0.2689414	0.5377348
**PTTG3**	0.60056764	-2.9629462	-0.3696618	0.12476157	0.76906383
**NBN**	0.36876827	-2.956436	-0.20860624	0.070560046	0.8621067
**NMI**	0.3975871	-2.9504156	-0.60336685	0.20450233	0.63301134
**LMNB1**	0.035099853	-2.9482322	-0.6730685	0.22829562	0.6016637
**NA**	-0.37895456	-2.946444	-0.55527735	0.18845677	0.64102256
**NFKBIA**	0.38673994	-2.9453695	-0.35041523	0.11897157	0.79686767
**WARS**	1.410312	-2.9430494	-1.1306973	0.3841924	0.36755747
**MSL3L1**	0.34310973	-2.9332507	-0.23070955	0.0786532	0.84890777
**NA**	-0.1366471	-2.9313598	-0.26527834	0.09049668	0.82100743
**CYP26A1**	-0.74883944	-2.9243166	-0.9719424	0.33236566	0.53781015
**OBFC2A**	0.427519	-2.9228666	-0.7183533	0.24577013	0.5543896
**NA**	-0.08248609	-2.922572	-0.22222137	0.07603624	0.8518171
**IFIT1**	-0.06435794	-2.921479	-1.1730065	0.4015112	0.3474283
**AIM2**	-1.7190115	-2.9182653	-0.47119045	0.16146252	0.73248047
**LOC652694**	0.20519401	-2.9135387	-1.0439568	0.3583123	0.41600358
**YEATS2**	1.5121676	-2.90025	-0.36150312	0.12464551	0.7614019
**LOC26010**	0.047647182	-2.8996515	-0.71855545	0.24780752	0.5185895
**PLAC8**	0.5129178	-2.89236	-0.823555	0.2847346	0.5947455
**CD276**	-0.9205053	-2.8922212	-0.13680553	0.0473012	0.9081713
**SUCNR1**	0.95365775	-2.8911238	-0.23179197	0.08017366	0.8430079
**IRF7**	-0.039733853	-2.8893933	-0.9255152	0.3203147	0.4855806
**CD274**	-0.920663	-2.8890505	-0.5298605	0.18340299	0.639894
**IL1F7**	-0.05379533	-2.886481	-0.8392725	0.29075974	0.5299799
**TNFRSF9**	1.1212257	-2.8831277	-0.39218664	0.13602819	0.75422376

**PSMB8**	0.5812234	-2.870589	-0.5471363	0.19060071	0.6893601
**EZH2**	-0.6126608	-2.8692281	-0.41121006	0.14331731	0.7369426
**PELI1**	0.48076856	-2.8682275	-1.3183756	0.4596482	0.44151935
**AQP9**	-1.4782128	-2.8666112	-1.493433	0.52097505	0.42546812
**BATF3**	-1.2814198	-2.8593025	-0.9640889	0.33717626	0.5655858
**CMPK2**	-0.828136	-2.8584173	-0.84870195	0.29691324	0.49515393
**ZKSCAN2**	1.6314819	-2.8583496	-0.3025174	0.1058364	0.80914736
**SAMD4A**	0.7304414	-2.8533995	-0.6032939	0.21142988	0.61429363
**SP110**	0.89064187	-2.8530369	-0.4809413	0.16857171	0.6897933
**CCL5**	-0.9393529	-2.8444417	-0.4135027	0.14537218	0.761383
**UBE2J1**	1.270717	-2.842738	-0.45941067	0.16160852	0.75224686
**DNAJA1**	-0.6976562	-2.8344507	-0.60028076	0.21178028	0.65540725
**MSL3L1**	0.34319794	-2.8338985	-0.2962594	0.10454129	0.8121099
**CDKN3**	-0.88744575	-2.8259933	-0.21701097	0.07679104	0.853511
**UBE2C**	1.2628146	-2.8215623	-0.3749051	0.13287146	0.74337333
**PI4K2B**	0.49821132	-2.811212	-0.7919054	0.28169537	0.48544598
**OASL**	0.42685512	-2.8059611	-1.626173	0.5795422	0.33726895
**DMXL2**	-0.69850266	-2.8039422	-0.4635954	0.165337	0.72824186
**LOC647450**	0.15991324	-2.7987826	-1.1905851	0.42539394	0.4474752
**TNFAIP2**	1.1145047	-2.7965589	-0.8010187	0.28643012	0.590673
**NA**	-0.26471645	-2.79476	-0.35224247	0.12603675	0.7554469
**HERC6**	-0.43410146	-2.7904859	-0.7371421	0.26416263	0.5163029
**IL15RA**	-0.055787712	-2.788209	-0.588109	0.21092717	0.62359124
**MCOLN2**	0.29249647	-2.7840424	-0.6891155	0.24752337	0.5741496
**RIN2**	0.6676488	-2.7824187	-1.4597206	0.5246229	0.40066493
**MGC39372**	0.3095949	-2.7766402	-1.07199	0.3860745	0.45362768
**CH25H**	-0.8659307	-2.7751105	-1.2173233	0.4386576	0.2958307
**UBE2L6**	1.2728497	-2.7749603	-0.72933674	0.2628278	0.6526403
**DUSP5**	-0.6712507	-2.7710962	-1.0294533	0.37149677	0.5710575
**GRAMD3**	-0.46445507	-2.7702954	-0.44886494	0.16202782	0.71274096
**HIST1H4K**	-0.42433366	-2.7699432	-0.34911346	0.12603633	0.7857145
**HIST1H2BN**	-0.42585063	-2.768297	-0.1362729	0.04922626	0.9082275

**GINS2**	-0.49376842	-2.765965	-0.42301178	0.15293461	0.7148175
**BPGM**	-1.2295699	-2.764425	-0.386086	0.13966231	0.74483824
**CDC45L**	-0.8981064	-2.7634597	-0.4356451	0.15764482	0.7001552
**HERC5**	-0.43419352	-2.7576168	-1.6692114	0.6053094	0.2874709
**C11orf75**	-1.1775552	-2.757115	-0.5849128	0.21214668	0.64473075
**APOL6**	-1.4878733	-2.7534676	-0.49040222	0.1781035	0.6653048
**NCAPG**	0.37095085	-2.7506695	-0.2678547	0.097378	0.81766313
**TRIM21**	1.1692727	-2.7364476	-0.6540365	0.23900934	0.59627193
**CCNB2**	-0.93751544	-2.7305439	-0.38956213	0.14266834	0.74407065
**OXSR1**	0.44805205	-2.7274585	-0.42287827	0.15504481	0.73770237
**LOC441019**	0.08445239	-2.722548	-0.6378555	0.23428623	0.6014139
**NA**	-0.40303454	-2.7220407	-0.6016073	0.22101335	0.6181926
**CASP7**	-0.9724967	-2.715913	-0.51755285	0.19056311	0.6649754
**MR1**	0.32889488	-2.7158763	-0.42372656	0.15601836	0.7217215
**C15orf48**	-1.142984	-2.7158601	-1.3522272	0.49790016	0.45400542
**STAT2**	0.93654346	-2.7146766	-0.7467108	0.27506435	0.5976397
**CFLAR**	-0.8675699	-2.7131095	-0.5594406	0.20619905	0.68020916
**IFNG**	-0.062456194	-2.7105718	-1.2162476	0.44870517	0.44437358
**NLRC5**	0.39460063	-2.7065458	-0.42261314	0.15614483	0.76349324
**CASP4**	-0.97354424	-2.7049656	-0.47670078	0.17623174	0.7118474
**SLC43A3**	0.8365588	-2.7026854	-0.3796525	0.14047232	0.74895483
**TK1**	1.0537102	-2.7023575	-0.3347106	0.12385874	0.7746922
**EIF2AK2**	-0.64998466	-2.7007682	-0.9589453	0.3550639	0.45914164
**LOC643997**	0.12164942	-2.699231	-0.29249477	0.108362265	0.8085494
**MGC29506**	0.30818275	-2.6942391	-0.7766876	0.28827718	0.4883514
**MXI1**	0.3548203	3.336374	0.21884489	0.06559363	1.1678367
**CXXC5**	-0.75460875	3.355098	0.75873184	0.22614297	1.6827801
**GLTP**	-0.48851326	3.374232	0.52899647	0.15677537	1.4240466
**CTDSP2**	-0.7726914	3.3758397	0.69990253	0.20732695	1.6576083
**LOC653103**	0.20998333	3.383266	0.37982464	0.112265676	1.3129019
**LOC730316**	0.24280547	3.386709	0.4444132	0.13122272	1.3697579
**HBEGF**	-0.4426067	3.3967972	1.402669	0.41293868	2.8503883

**TSPAN4**	1.2053695	3.5200386	0.56306267	0.15995923	1.4950634
**PTDSS1**	0.58929396	3.537718	0.4015255	0.11349844	1.3212758
**MRPL45**	0.33522627	3.5413284	0.3730712	0.10534781	1.2918046
**ZNF34**	1.7846655	3.561116	0.35599804	0.09996811	1.2875334
**CABC1**	-0.99779123	3.566188	0.5301933	0.14867228	1.4451729
**VEGFB**	1.3720226	3.657623	0.5577736	0.15249619	1.4445028
**ZDHHC7**	1.5936121	3.6603582	0.7073345	0.19324188	1.7052039
**MEF2D**	0.2981564	3.6701992	0.6924658	0.18867254	1.5831358
**PIK3IP1**	0.5036851	3.7065449	0.6473732	0.17465678	1.5731415
**ARRDC2**	-1.4073013	3.8193815	0.5582261	0.14615615	1.4492002
**TOB1**	1.1313546	3.9774272	0.90807724	0.2283077	1.975464
**ING1**	-0.047008947	4.077989	0.46914577	0.11504341	1.4024422
**AXIN2**	-1.3070259	4.126832	0.5361223	0.12991135	1.4615514
**FOXJ2**	-0.530525	4.7123027	0.5525303	0.117252715	1.4846911
**KBTBD11**	-0.027755639	4.726356	0.665823	0.14087449	1.5950197

- Down-regulated gene; No sign- Up-regulated gene
