# Supplementary figures and images for: Genome-wide analysis of primary peripheral blood mononuclear cells from HIV + patients-pre-and post- HAART show immune activation and inflammation the main drivers of host gene expression
Source: Mol Cell Ther. 2014 Apr 3;2:11. doi: 10.1186/2052-8426-2-11 (PMC4451969; doi:10.1186/2052-8426-2-11)

## Additional file 1

### MetaCore™ pathway legend pictures

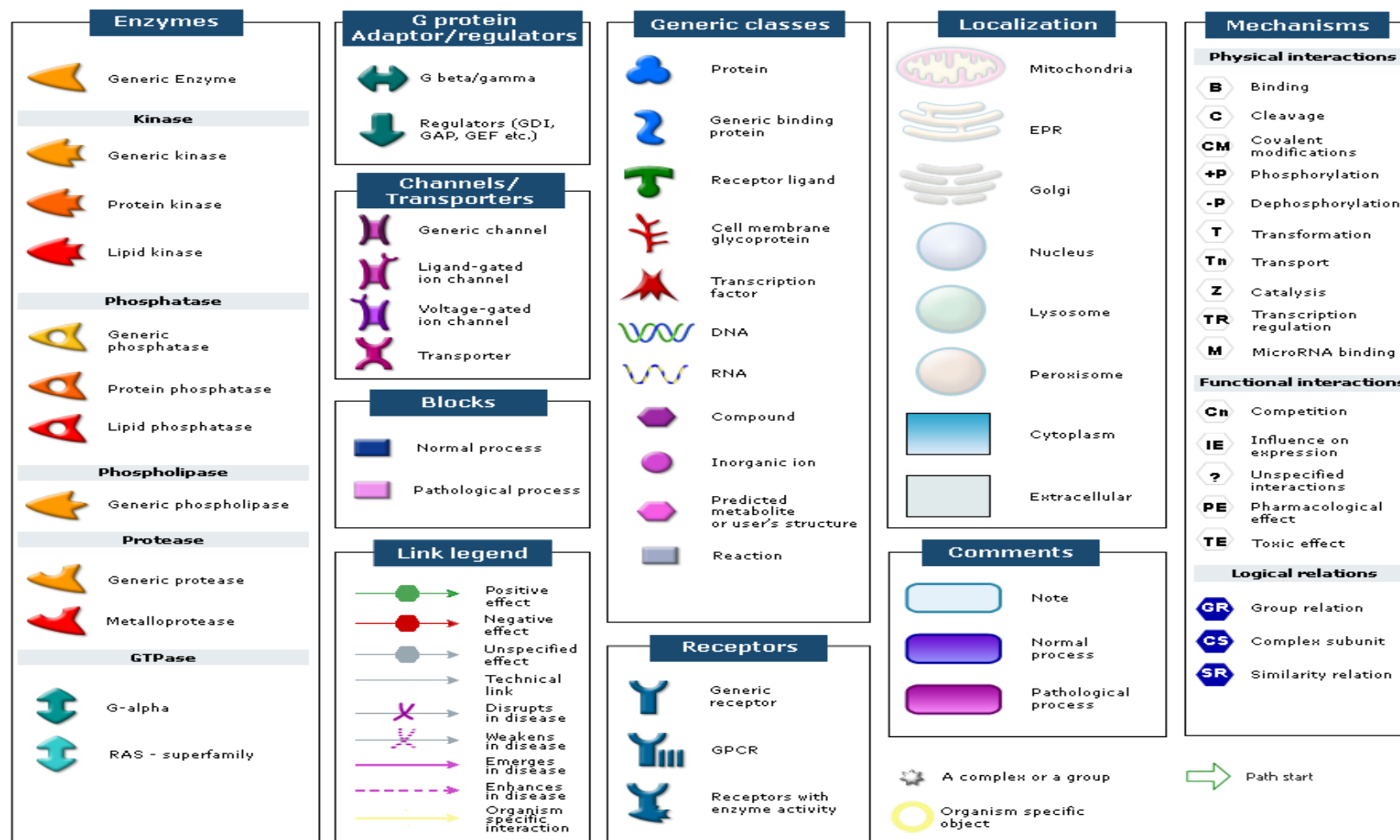

Supplement: Supplementary file 2 — Additional file 2: MetaCore™ pathway legend pictures. (PDF 185 KB) [file 40591_2013_14_MOESM2_ESM.pdf]

### Additional file 3

32 map folders corresponding from the comparison between TP1 and TP2

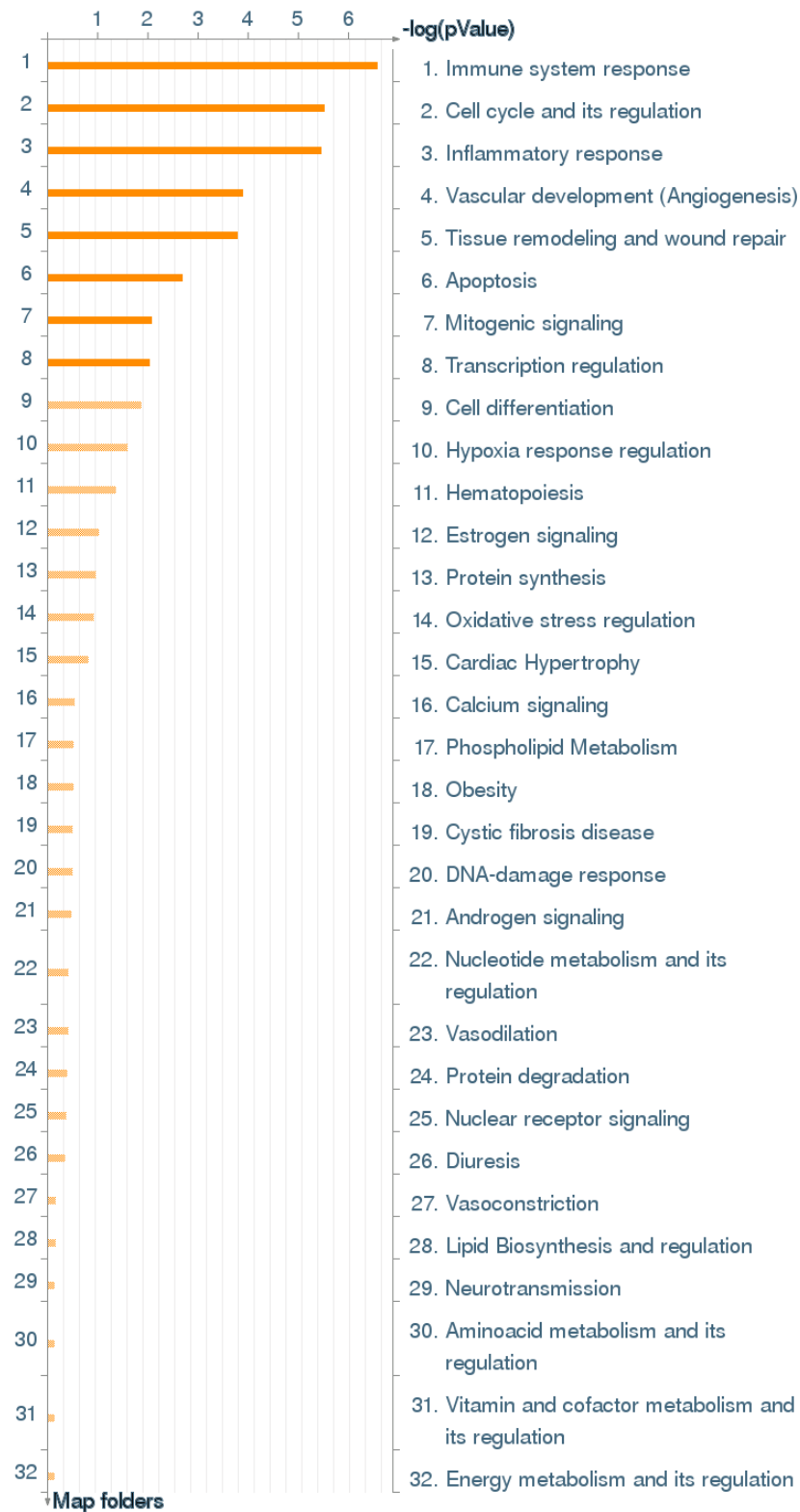

Supplement: Supplementary file 3 — Additional file 3: 32 map folders corresponding from the comparison between TP1 and TP2. (PDF 180 KB) [file 40591_2013_14_MOESM3_ESM.pdf]
