# Supplementary material for: Genome-wide analysis of primary peripheral blood mononuclear cells from HIV + patients-pre-and post- HAART show immune activation and inflammation the main drivers of host gene expression
Source: Mol Cell Ther. 2014 Apr 3;2:11. doi: 10.1186/2052-8426-2-11 (PMC4451969; doi:10.1186/2052-8426-2-11)
Supplement: Supplementary file 4 — Additional file 4: Pathway maps of the comparisons between TP1 versus TP2. (PDF 132 KB) [file 40591_2013_14_MOESM4_ESM.pdf]

Additional file file 4

Pathway maps of the comparisons between TP1 versus TP2

Maps	pValue	Ratio
Immune response_Antiviral actions of Interferons	4.325E-17	14/52
Immune response_IFN alpha/beta signaling pathway	7.557E-13	9/24
Immune response_Role of PKR in stress-induced antiviral cell response	1.727E-10	1057
Immune response_HMGB1/TLR signaling pathway	5.168E-08	736
Development_PEDF signaling	4.789E-07	7/49
Immune response_Innate immune response to RNA viral infection	7.313E-06	5/28
Immune response_HMGB1/RAGE signaling pathway	1.325E-05	6/53
Apoptosis and survival_Role of PKR in stress-induced apoptosis	1.325E-05	6/53
Transcription_Role of AP-1 in regulation of cellular metabolism	3.453E-05	5/38
Immune response_MIF-mediated glucocorticoid regulation	5.958E-05	4/22
LRRK2 and immune function in Parkinson's disease	5.958E-05	4/22
Immune response_Bacterial infections in normal airways	1.327E-04	5/50
Immune response_Role of HMGB1 in dendritic cell maturation and migration	1.376E-04	4/27
Immune response _IFN gamma signaling pathway	1.919E-04	5/54
Immune response_TLR signaling pathways	1.919E-04	5/54
Bacterial infections in CF airways	2.695E-04	5/58
Development_Angiotensin signaling via STATs	2.714E-04	4/32
Immune response_TREM1 signaling pathway	2.921E-04	5/59
Immune response_MIF in innate immunity response	6.490E-04	4/40
Apoptosis and survival_Inhibition of ROS-induced apoptosis by 17beta-estradiol	9.352E-04	4/44
Immune response_IL-1 signaling pathway	9.352E-04	4/44
Immune response_Inhibitory action of Lipoxins on pro-inflammatory TNF-alpha signaling	1.107E-03	44/46
Cell cycle_Chromosome condensation in prometaphase	1.130E-03	3/21
Immune response_IL-15 signaling via JAK-STAT cascade	1.483E-03	3/23
Immune response_Histamine signaling in dendritic cells	1.516E-03	4/50
Immune response_HSP60 and HSP70/ TLR signaling pathway	2.020E-03	4/54
Immune response_Antigen presentation by MHC class I	2.646E-03	3/28
Immune response_CD137 signaling in immune cell	2.930E-03	3/29
Immune response_IL-15 signaling	3.762E-03	4/64

**Development_PDGF signaling via STATs and NF-kB**	3.892E-03	3/32
**Apoptosis and survival_Caspase cascade**	4.251E-03	3/33
**Development_NOTCH1-mediated pathway for NF-KB activity modulation**	4.629E-03	3/34
**Development_Angiopoietin - Tie2 signaling**	5.026E-03	3/35
**Inhibitory action of Lipoxin A4 on PDGF, EGF and LTD4 signaling**	5.026E-03	3/35
**Immune response_Lipoxins and Resolvin E1 inhibitory action on neutrophil functions**	5.026E-03	3/35
**Influence of low doses of Arsenite on Glucose stimulated Insulin secretion in pancreatic cells**	5.444E-03	3/36
**Development_Lipoxin inhibitory action on PDGF, EGF and LTD4 signaling**	5.444E-03	3/36
**Inhibitory action of Lipoxins and Resolvin E1 on neutrophil functions**	7.320E-03	3/40
**Immune response_Th1 and Th2 cell differentiation**	7.320E-03	3/40
**Immune response_HMGB1 release from the cell**	7.842E-03	3/41
**Apoptosis and survival_TNFR1 signaling pathway**	8.950E-03	3/43
**Apoptosis and survival_FAS signaling cascades**	9.536E-03	3/44
**IL-1 beta-dependent CFTR expression**	1.085E-02	2/16
**Immune response_Histamine H1 receptor signaling in immune response**	1.210E-02	3/48
**LRRK2 in neuronal apoptosis in Parkinson's disease**	1.221E-02	2/17
**Development_G-CSF signaling**	1.280E-02	3/49
**Mucin expression in CF via TLRs, EGFR signaling pathways**	1.352E-02	3/50
**Apoptosis and survival_Endoplasmic reticulum stress response pathway**	1.582E-02	3/53
**Immune response_CCL2 signaling**	1.663E-02	3/54
**Cell cycle_Sister chromatid cohesion**	2.008E-02	2/22
